# Inside Their Minds: A Multi-Institutional Exploration into the Decision-Making of Medical School Competency Committee Members

**DOI:** 10.5334/pme.2361

**Published:** 2026-01-29

**Authors:** Michael S. Ryan, Pim W. Teunissen, Andrew S. Parsons, Elizabeth Bradley, Sally A. Santen, Anita V. Shelgikar, Daniel J. Schumacher, Matthew Kelleher, Shahab Jolani, Christina M. Vitto, Alexandra H. Vinson, For the UME Competency Committee Collaborators

**Affiliations:** 1 Department of Pediatrics, University of Virginia, Charlottesville, VA, USA; 2 Maastricht University, Maastricht, The Netherlands; 3 Department of Gynecology, Maastricht University, Maastricht, The Netherlands; 4 Department of Medicine, University of Virginia, Charlottesville, VA, USA; 5 Department of Medical Education, University of Virginia, Charlottesville, VA, USA; 6 Department of Emergency Medicine and Medical Education, University of Cincinnati, Cincinnati, OH, USA; 7 Department of Neurology University of Michigan Medical School, Ann Arbor, Michigan, USA; 8 Department of Pediatrics, University of Cincinnati College of Medicine, Cincinnati, Ohio, USA; 9 Departments of Internal Medicine and Pediatrics, University of Cincinnati College of Medicine, Cincinnati, Ohio, USA; 10 Department of Methodology and Statistics, Maastricht University, Maastricht, the Netherlands; 11 Departments of Emergency Medicine and Internal Medicine, Virginia Commonwealth University School of Medicine, Richmond, Virginia, USA; 12 Department of Learning Health Sciences, University of Michigan, Ann Arbor, Michigan, USA

## Abstract

**Introduction:**

A competency committee is a group of experts who make a consensus-based judgement about a learner’s competence. While evidence-based practices for post-graduate committees have been described, research and standards are lacking in undergraduate medical education. Medical school competency committees often distribute student reviews to individual members; therefore, understanding how they interpret assessment data is critical.

**Methods:**

Approaching our investigation from a social constructionist orientation, we conducted think aloud interviews with 22 competency committee members at 7 medical schools in the United States from 2023–2025. Participants were tasked with reviewing local student assessment data in preparation for a competency committee meeting and verbalizing thoughts to an investigator. We analyzed transcripts using an interpretivist approach, with sociocultural cognition theory as the primary interpretive lens.

**Results:**

Two major concepts depicted how participants processed student assessment data to arrive at competency determinations: contextual influences and internal reasoning processes. Contextual influences (i.e. goals, data available, and standards for interpreting the data) were outside the direct control of participants. Internal reasoning processes included: first impressions, interpreting trends, and negotiating conflicting data. Contextual influences varied and served as the lens through which participants interpreted assessment data.

**Discussion:**

This study provides the first examination into the thought processes used by medical school competency committee members to make their decisions. Participants used decision-making strategies that parallel those observed in other cognitive reasoning tasks. Contextual influences were foundational in how participants interpreted assessment data, highlighting how competence is socially constructed.

## Introduction

Competency committees provide consensus-based judgements of a learner’s progression toward competent clinical practice and are critical to the successful delivery of competency-based medical education (CBME) [1,2]. A competency committee is defined as a group that works collaboratively to determine a learner’s level of entrustment or competency using aggregate assessment data [1,2]. In post-graduate medical education, evidence-based practices for competency committee operations have been well-described [3,4,5,6], and in many countries, such committees are now mandated for post-graduate accreditation [3,7,8]. Recently, international leaders in undergraduate medical education have embraced CBME [9,10,11], and some have explored the implementation of a competency committee structure [11,12,13].

Though some approaches used in post-graduate settings may translate to undergraduate medical education, features such as the medical students’ limited role in clinical practice [14], large learner volumes [15], and stress related to the transition into residency [16], suggest the medical school environment may require unique considerations. Furthermore, unlike their counterparts in post-graduate training, medical school accrediting bodies in many Western countries have not mandated competency committees. Most undergraduate medical education accreditors require that schools make decisions regarding a learner’s advancement and/or graduation using a common set of processes and standards [17,18,19]. As a result, medical school advancement committees focus primarily on deficiencies [20] and organize their work around whether students have met academic performance metrics (i.e. passing tests and/or courses) [17,18,19].

Recent data indicates that only 18.7% of medical schools in the United States (US) use a competency committee model; the majority maintain a more traditional advancement committee model [21]. However, interest in medical school competency committees has increased in recent years [12,13,22,23,24]. In our previous work, we examined how seven innovative US medical schools designed their competency committees [25]. Similar to post-graduate programs [2], the undergraduate medical education programs in our study distributed labor to subgroups or individuals to manage the logistics associated with reviewing large volumes of learners. In many cases, individuals were afforded significant responsibility for triaging which students would require presentation to the larger competency committee [25]. Past research into medical school competency committee operations has focused almost exclusively on the group decision-making of the larger competency committee [5,26,27,28]. Several studies investigated the perspectives of committee members [11,28,29,30], though only two included members of medical school committees [11,29], and none have directly investigated how individuals made decisions outside the larger competency committee.

An understanding of how individual medical school competency committee members come to competency determinations is necessary to support a defensible summative decision. Therefore, we sought to investigate the thought processes used by competency committee members when tasked with making competency decisions on medical students. Through this exploration, we aimed to characterize how medical school competency committee members come to their decisions regarding student competence (or incompetence).

## Methods

### Theoretical Framework

We frame the work of medical school competency committee members as an information processing task, where the work involves formulating a decision as to the fitness of a student into socially constructed categories of ‘competent’ or ‘not competent.’ As such, we draw on sociocultural cognition theory, a family of theories that describe the relationship between human cognition and culture [31]. A common environment can stabilize cognitive processes across individuals, producing people who ‘think alike’ or engage in sensemaking together [32]. Shared schemas developed through common socialization processes facilitate collective sensemaking by organizing cognition [32]. Schemas can also be cultural models that carry with them normative expectations, meaning that schemas can be used in evaluative contexts [33]. Within these schemas, members employ scripts, consistent with script theory [34], which are organized knowledge structures describing, for example, what a competent or not competent student ‘looks like.’

### Paradigm and Approach

We approached this qualitative, multi-institutional, concurrent think aloud interview study [35] from a social constructionist paradigm [36], reflecting our view that competence is a socially-constructed phenomenon [37]. Within this definition, we view competency decisions as inherently context-dependent, influenced by the environment and culture in which the learner and supervisor practices [17].

Think aloud interviews are a mechanism for accessing, through verbalization, working memory around complex thought processes such as judgement and problem-solving [38]. Think aloud interviews can be further characterized as concurrent (during a task) or retrospective (following a task). We conducted a concurrent think aloud to gain insight into the cognitive processes underlying the task of making competency judgements. We selected a concurrent approach to minimize retrospective accounting bias [38]. The task involved real-time interpretation and verbalization of assessment data for several students whom the competency committee member was required to review in preparation for an upcoming competency committee meeting.

### Setting

This study was conducted between November 2023 and April 2025. At the time of the study, we were aware of only 7 US-based MD programs that had a “mature” competency committee. By mature, we mean that the competency committee had been implemented for at least 3 years. We felt mature programs would represent relatively stable processes for reviewing student files.

A summary of sites included in the study is presented in Table 1. As demonstrated, sites varied by their committee size, workload distribution, and which students were presented to the larger competency committee. All members of the site’s competency committee were eligible for participation. Depending on institutional ethics requirements, either the lead investigator (MSR) or the local committee chair contacted committee members through email and/or in-person communication. Up to three requests were sent. Interest was indicated by email to the lead investigator or an online sign-up sheet. This study was approved or deemed exempt by each participating institution.

**Table 1:** Description of undergraduate medical education competency committees and their respective workload distributions for a multi-institutional study of competency committee members in the US (n = 22, 2023–2025).


INSTITUTION	COMMITTEE ESTABLISHED (YEAR)	CLASS SIZE^a^ (n)	COMMITTEE SIZE (n)	WORKLOAD DISTRIBUTION	PRESENTATION TO LARGER COMPETENCY COMMITTEE

Oregon Health & Science University	2017	150	22	The student body is distributed to committee member pairs.	Only when disagreements are present.

University of Cincinnati	2021	177^b^	14	All members review all students.^b^	All students are presented.

University of Michigan	2016	164	10	Student body is divided into cohort groups; competency committee pairs review each student; all members review students with concerns.	All students are presented periodically; attention is focused on those with concerns.

University of Minnesota	2021	241	31	Students are divided into 12 subcommittees (3 members each); subcommittee members review each student in their cohort.	Never; subcommittees function independently.

University of Virginia	2018	158	16	Class is divided into 3 subgroups (3 members each).	When disagreements are present and/or when students are identified with concerns.

Virginia Commonwealth University	2019	184^b^	25	All members review all students.^b^	All students are presented.

Washington University	2020	124	11	Student assessment data is divided by competency domain; 3 members review 2 domains each.	Subcommittees provide notes on all students and formal reports on students with concerns.


^a^ Data based on AAMC matriculant data from 2023–2024. Class sizes vary to some extent year-to-year [37]. ^b^The competency committee makes decisions on a small cohort (approximately 5–10/class) of students at these institutions.

### Researcher characteristics and reflexivity

Our research team was assembled to provide a balance of perspectives and experiences, including those with intimate knowledge of competency committee operations and those without. Four authors served as primary coders (MSR, EB, AVS, AHV). The primary investigator (MSR) identifies as a pediatrician, clinician educator, and educational researcher with particular interest in CBME. His perspective was influenced by experience as a physician trainee in the US, previous service on undergraduate and post-graduate competency committees, and involvement in a national CBME pilot program. EB is a medical educator, program evaluator, and qualitative researcher. She drew upon her experience serving as a member of a medical school competency committee for several years. AVS is a neurologist and educational program leader. Her experience on a post-graduate competency committee and more recent experience as a new medical school competency committee chair informed her perspective. AHV is a sociologist and social science researcher. Her perspective was informed by post-doctoral ethnographic studies including an in-depth investigation of professional socialization of the physician role in patient encounters in US medical training. Additional members of the author team included those who serve/served as members of competency committees in the US (ASP, SAS, DJS, MK, CMV) and those without previous roles in competency committees in any setting (PWT, SJ). The entire study team met monthly to discuss initial findings, bringing forward their perspectives as competency committee leaders, clinician educators, and education researchers.

### Data collection methods and instruments

#### Overview and background data collection

One-on-one interviews were conducted by one investigator (MSR) using an online platform. Interviews were audio/videorecorded and transcribed verbatim. Following a study overview and consent, the interviewer first gathered background information to understand the participants’ competency committee experience and, in general, the process they used to review student files.

#### Think aloud

We conducted concurrent think aloud interviews using a process outlined by Johnson and colleagues [35]. Participants were instructed to access their local dashboard/portfolio system to identify real student(s) whom they were required to review. Participants shared their screens virtually with the investigator if permitted by the local institutional review board and/or institutional policy. To preserve the authenticity of the task, the interviewer asked participants to follow their normal review procedures, verbalizing their internal thought processes throughout. He requested that participants conduct several reviews in real-time, aiming for 2–3 student reviews per participant. Finally, the interviewer asked questions generated from his field notes. The purpose of this final step, referred to as retrospective reporting in think-aloud methodology [38], was to add clarity and trustworthiness to the findings. The interview guide is provided in the **Supplemental Appendix**.

### Data analysis

We conducted our analysis from an interpretivist approach [35], reflecting our perspective that the thought processes used by competency committee members are inherently centered on meaning-making from information available and the contextual framing of the review task. Two coders (MSR and AHV) first read one transcript from each institution (n = 7) to familiarize themselves with the data. Next, they independently coded each of these seven transcripts, convening weekly to develop a preliminary set of codes and definitions. The preliminary codebook was shared with two additional coders (EB and AVS), and collectively, the group of four investigators coded the remainder. This team met on a bi-monthly basis to reconcile codes until a final codebook was assembled.

As we proceeded with coding, we felt as though the interviews were portraying internal decision-making schemas used by participants. Thus, we began to focus our interest on the interaction between schemas (as broader decision-making frames) and scripts (as specific, pattern-matched prototypes), incorporating sociocultural cognition theory [31] and, specifically, script theory [34] as the primary lens for our interpretation. The critical importance of situated cognition [39], and the context specificity of competence [37,40,41], also became apparent. Through continued re-engagement with the literature, the data, and iterative discussions with the larger authorship team, we refined our interpretation of the findings into their final form.

## Results

A total of 22 interviews were conducted, from which 55 discrete student cases were discussed. Most participants (n = 14, 64%) were women, and all but two (n = 20, 91%) were clinician-educators who served as both members of the competency committee and front-line faculty supervisors in the clinical environment.

**Figure 1:**
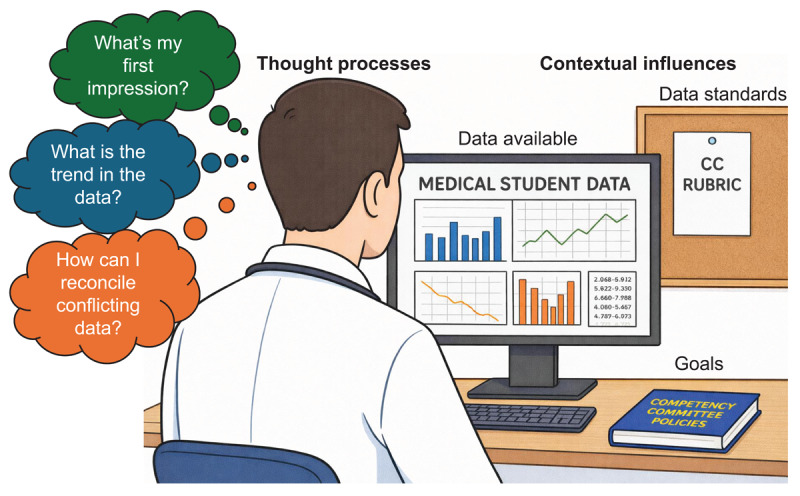
The relationship between contextual influences and thought processes used by undergraduate medical education competency committee members to determine student competence.

We identified two major concepts that depict how participants processed student assessment data: *contextual influences* and *internal reasoning processes*. Contextual influences refer to environmental factors outside the direct control of the participant’s thought processes. Internal reasoning strategies refer to the specific thought processes committee members used to work through assessment data. The relationship between these concepts in is illustrated in Figure 1 [42].

### Contextual influences

Contextual influences served as the lens by which assessment data was processed. These influences were intimately related to the setting and/or culture of the competency committee practice and included the goal of the review and the data/data standards used. Goals provided an overarching purpose for the review while the data and data standards provided specific details for how assessment data could be translated into a decision.

**Goal of the review**. There were three different goals for reviews: determining which students met criteria for *competence*, determining which may meet criteria for *incompetence*, or judging the student’s overall progress. Importantly, when participants focused on criteria for competence or incompetence, it was truly an *either/or* phenomenon. In other words, some participants described a process in which they were searching strictly for positive performance indicators, asking essentially, “is this student competent?” For example:

Let me see those (workplace-based assessments). So, one is neurology, it’s a resident, and then family medicine, we have two okay. So, it’s basically only three rotations, so (she) doesn’t meet criteria (for competency). (G1)

In contrast, other participants described a similar process, but with the opposite goal, essentially asking “is this student (potentially) *incompetent*?” The following provides a quotation from a participant who exemplified this goal:

So, for [this competency], for example, I can see […] I can just scale this down to [this] cutoff [score] [*shows filtering for specific score*][…] And now I can just see that there’s only three students that applies to. And then we can look at those students in detail and see what’s up with that. (B3)

Finally, some participants described a more developmentally oriented approach in which the goal was to determine the *degree to which* each student was on track toward attaining competence. As one participant shared, “we are trying to give guidance to the students […] for further reflection, further improvement, [to] guide their improvement.” (A2). When participants favored this approach, their reviews took on a more nuanced perspective – identifying areas of strength and improvement for *each* student which were then shared to students and coaches.

**Standards**. The second major contextual influence involved the data available and standards for how that data could be interpreted. Some participants were provided only workplace-based assessment data, while others incorporated data from other assessments such as multiple-choice tests, reflective writing assignments, and professionalism evaluations. From these data sources, local standards were used to interpret results, thus providing participants with guidelines for operationalizing competence in their respective context. Standards appeared indirectly related to the goals of the committee. For example, when goals were centered on determining if students met criteria for competence or incompetence, participants described more formal and rigid criteria. When goals were centered around progress, standards were less formal and less stringent.

In some cases, participants described a top-down, explicit, and rules-based threshold for competency (or incompetency). These standards were highly quantitative and were inclusive of a wide variety of assessments, from multiple-choice examinations to workplace-based assessments. Importantly, thresholds varied from participant to participant. For example, one participant might describe a threshold of 70% on all pre-clinical examinations, while another would describe a threshold of 75%. Standards were non-negotiable, and therefore, the role of the competency committee member was to verify whether the student met established criteria.

There are all these rules, if a student fails (an examination), if their cumulative score in one of their preclinical blocks is less than 75%…they come to the [larger] competency committee. (F3)

So, I’ll go through, like this student, we’re looking for level fours, and I don’t have the rubric right in front of me, but it’s typically I think four level fours or something like that[…] But this review process, I just look to see if it’s strictly on WBAs, can I make a decision or not? So, (for) this student, I can’t. (G4)

Others described a more iterative process of developing standards, one in which committee members were involved. In these situations, standards developed organically, based on “what seemed reasonable,” (B1), however, the result was still a firm criterion endorsed by the competency committee member. For example, “I think that 3 [on a supervision scale] sort of became what [the standard] was.” (D3).

Conversely, some members reported little formality to the standards. Though these participants suggested some degree of comparability to other members of their local committee, they embraced the inherent subjectivity of the review process.

They are meeting a minimum threshold in terms of what each of us *probably sets in our mind* [*emphasis added*] in terms of the quantitative data to say, yeah, they’re probably at a point where they would be set up for success in the next stage of their training. (A1)

Participants commonly pointed toward faculty development efforts that informed how standards were instilled. Those who endorsed formal approaches referred to rubrics they were instructed to use to guide reviews while those with less formal procedures cited discussions with their competency committee chair and/or other committee members as influencing their standards.

### Internal reasoning strategies

Contextual factors were highly variable and provided significant influence over *what* and *how* data were considered. However, the internal reasoning strategies were quite similar. Participants assembled judgements from a process that included: first impressions, evaluation of trends, and addressing conflicting data.

**First impressions**. Upon opening a student file, participants were presented with a summary that included numerical means, visual representations (e.g. graphs or figures), or other summary indicators of performance, often with comparisons to other members of the cohort. From these representations, they would “get the vibe[s]” (E3) of the student’s performance and formulate a first impression.

I look at this [*pulls up a summary document*] first to get a sense […] so, I just get a general sense of where they are at, for their average, and visually with these bubbles [*figure depicting mean scores*]. (A2)

The first impression could serve as a mechanism for triage, “I make a note of these performers here […] because they are below our threshold,” (B2) or more generally, to offer a tentative categorization of the student as ‘on track’ or ‘not on track’ toward achievement of competence. “I think the first thing that I’ll sort of look for is whether or not there are any red flags […], so that’s kind of where I’ll start, and the really looking at the rest of the EPAs and kind of where the majority of the ratings are falling.” (A1).

**Evaluation for trends**. Once an initial impression was formulated, participants sought confirmation. Trends were particularly important: “I’m taking a glance at their performance on exams, and you can see, they started out okay in [course] then they sure didn’t do all that great in [course]… so I’m noting it just trends over time,” (B2).” Trends came with an implied expectation for what constitutes ‘normal’ progression. An upward trajectory was particularly important and added confidence that a student was progressing. For example:

I look at the general trend. This is the shape [*points toward scores that are increasing over time*] that I’m expecting to see. So, I do kind of look at the shape and if there’s weirdness, I notice that. (E3)

**Addressing conflicting data**. Participants frequently encountered conflicting data. Conflicts involved discrepancies between individual raters and/or rater types (e.g. attending physicians versus residents), higher versus lower scores, and misalignment between numerical scores and narratives. The perspectives of attending physicians were trusted more than junior supervisors because “attendings are more experienced in doing all of this than residents and fellows.” (D2). Personal knowledge of the assessor weighed heavily in instilling trust in individual data points, “she’s one of our people […] so, I trust [NAME’s] rating.” (E3) Findings seemed discounted when the participant did not know the rater, for example:

I don’t know if this one evaluator just tends to put people at [this score] […] I’m unable to read between the lines because I don’t know who this evaluator is. (C1)

When conflicting data were present, committee members pursued a more detailed review to assist in reconciliation. Of note, there was acceptance for some sort of deviation, “if there’s a few blips along the way, I’m not quite as concerned.” (C2) and participants frequently provided explanations for deviations which were framed as anomalous, i.e. “it was just sort of a bad day” (C3). Critically, deviations rarely changed their first impression, particularly when viewed as rare or inconsistent.

I’m struggling a bit, to be honest […] there’s things that don’t seem to line up as well with fives [on an entrustment scale], but it’s hard for me to push them lower because it’s somewhat consistent across contexts and they seem to have reasonably supportive comments. (E2)

Participants were frequently confronted by poor alignment between scores and comments. A pervasive concern was a relative distrust in a data obtained from the workplace. For example:

[…] and then the neurology exam was a 2.0 (on a scale of 1–4, where 1 is the lowest), but the comment is just, ‘Continue to examine as many patients as possible,’ And the comment is actually really good, so I don’t know if he knew what a 2.0 meant. (D1)

This often caused the committee member to litigate what was, the reality of the performance that they didn’t personally observe.

You’ll read a comment and you’ll be like, ‘wow, this person has really high expectations’ […] or on the other side of things, you see people get rated at a five because they’re like, ‘they’re performing where I expect them to,’ and I’m like, that’s not necessarily a five, you know […] so there’s a little more, I think, interpretation that goes into it. (E1)

## Discussion

This study explored the thought processes used by competency committee members at seven US medical schools. Previous studies, predominantly in post-graduate medical education, have examined the decision-making of competency committee members indirectly through interviews [11,29,43,44] and observations of committee deliberations [26,27,28,30,45]. The present study adds to the existing literature through direct exploration of medical school competency committee member decision-making, thus providing the first examination of this important sociocultural cognitive task in undergraduate medical education. Overall, our findings offer insight into thought process used by competency committee members and how contextual factors influenced their interpretation of competence. In this section, we examine the similarities between reasoning strategies identified in our study and clinical reasoning literature. We then explore the critical role context played in developing schemas and scripts for competent (and incompetent) students. Throughout, we offer implications of these findings for medical school competency committee practice.

### Insight into internal reasoning strategies: application from the clinical reasoning literature

The clinical reasoning literature warns of several cognitive biases that impact clinical decision-making processes [46]. Examples include: anchoring, framing, and confirmation biases [46];. For each, methods have been described to reduce their impact [47]. Parallels for many forms of cognitive bias are present throughout our findings, including the value of first impressions (i.e. anchoring bias), how data was represented (i.e. framing bias), how members handle conflicting data (i.e. confirmation bias). The observation of such parallels suggest that similar strategies for combating biases [46,47] may be necessary to ensure accuracy and equity in competency-based decision making.

Clinical reasoning literature also describes two decision-making methods commonly used in clinical practice: deductive and inductive decision-making [46]. Deductive methods are systematic and rely on well-defined standards [48], whereas, inductive methods rely on pattern recognition and individual expertise [46]. Novices tend to use deductive approaches to ensure consistency to a standard while experts begin inductively and shift toward deductive approaches to confirm their suspicions [46].

In our study, participants used a combination of inductive and deductive decision-making strategies. Inductive approaches were common when reviewing files; initial impressions triggered a working ‘diagnosis’ of student competence, which was then tested and refined as further evidence was accumulated in their reviews. However, this approach was filtered through a deductive lens, informed by contextual factors that operationalized the standard by which competency was viewed. Interestingly, the balance between inductive and deductive decision-making was more dependent on context-specific standards than expertise.

### The critical role of context in competency decision-making

Like other social constructs, competence is inherently context-dependent and defined by cultural norms and expectations [36,41]. ten Cate and colleagues recently proposed that some aspects of clinical competence are universal (context-independent) while others are setting-specific (context-dependent) [49]. Similarly, Durning and colleagues noted that one’s ability to practice competently is dependent on the context of the practice [50]. Hodges and Lingard elaborate on this notion extensively in *The Question of Competence* [41], posing many challenges to CBME, including fundamental concerns with universal standards for the construct [41]. Our results highlight the critical impact of context on competency committee members’ competency-decision making members. Local standards and norms were foundational in how each member reviewed student data and how competency was operationalized in practice.

**Figure 2:**
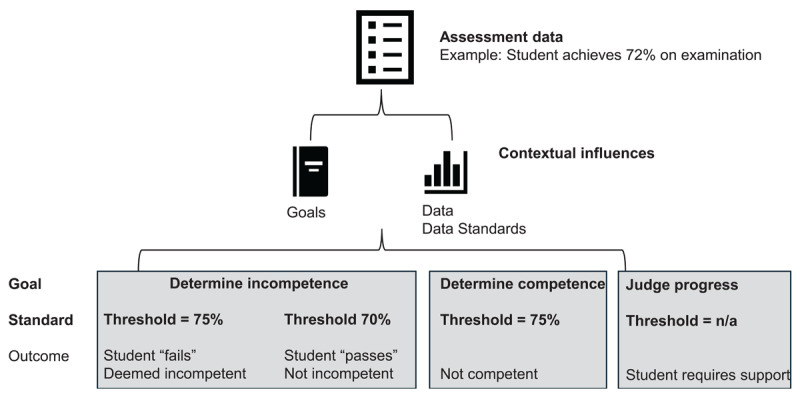
Illustration of how the same assessment data may be interpreted differently as a consequence of contextual factors.

These findings are highly consequential. To illustrate their significance, we may consider a hypothetical case of a student who receives a score of 72% on a standardized test administered in the clinical phase of their training (Figure 2). As demonstrated in our study, competency committee members practiced in highly variable contexts, with different goals, data sources, and standards for their interpretation. As such, the same score could have vastly different meanings from one committee member to the next.

These different methods for operationalizing competency raise a significant philosophical question: should we conceptualize competency attainment as the *presence of competency*, *absence of deficiency*, or overall progress and growth? Determining whether students meet criteria for competence or incompetence may offer a practical mechanism for implementing summative decision-making; trainees are *awarded* or *denied* increased autonomy on the basis of performance over time [51]. In contrast, assessing students for progress reflects a developmental mindset, one that previous authors have argued is critical to support the attainment of competency for all trainees and directing resources where most appropriate [22,44].

Perhaps most significantly though, the illustration raises an even more critical question. What does it mean if a student could receive the same score on the same examination, yet attain a completely different outcome? If the ultimate goal of CBME is to afford accountability to the public and the profession [52], is such aspiration achievable when the notion of competency is so heavily influenced by contextual factors? Our findings suggest challenges in how competency is conceptualized across diverse contexts, an issue likely to persist in the absence of mandates from medical school accrediting bodies.

### Limitations

There are several limitations to this study. First, participants represented seven US-based institutions and thus our findings may be limited to those with similarly structured competency committees within a similar context. Second, those who participated were volunteers and may not represent the diverse thought processes of the entire local committee. Third, the think aloud method has several established limitations including participant burden, artificiality of the verbalizing thoughts, and potential cultural influences [35,38]. Fourth, due to ethical considerations, we did not explore the identity of the learners under review and how those identities may have influenced the practices of participants. Finally, this study was designed to examine the practices of the medical school competency committee members during their review of student files and does not provide insight into the committee deliberations that followed. In our future work, we plan to observe the competency committees themselves to help triangulate our findings from this study and formulate a comprehensive picture of competency committee cultural practices.

## Conclusions

This study characterized how twenty-two competency committee members assessed the competence of medical students using aggregate assessment data. Our findings provide empiric evidence for how competence is conceptualized and how those understandings vary significantly by context. Overall, this study provides insight into the thought processes used by competency committee members in US medical schools which add to our growing understanding of the cultural practices of competency committees in the undergraduate medical education environment.

## Data Accessibility Statement

A summary of the data supporting the findings for this study may be available upon request. Verbatim data is not available due to ethical need to preserve anonymity of participants.

## Additional File

The additional file for this article can be found as follows:

10.5334/pme.2361.s1 Supplemental Digital Appendix 1. Think Aloud Interview.
