# Supplementary material for: Inside Their Minds: A Multi-Institutional Exploration into the Decision-Making of Medical School Competency Committee Members
Source: Perspect Med Educ. 2026 Jan 29;15(1):53–64. doi: 10.5334/pme.2361 (PMC12857619; doi:10.5334/pme.2361)
Supplement: Supplemental Digital Appendix 1. — Think Aloud Interview. [file pme-15-1-2361-s1.pdf]

Supplemental Digital Appendix 1
Think Aloud Interview

Background Information/Overview:
Role in competency committee:
Time serving in competency committee:
Interest in competency committee: What drew you to participate in the competency committee?
General approach to file review: How do you approach your review of students?

Cases:
Instructions:
1. Open dashboard (or similar)
2. Share screen (if permissible)
3. Identify a student whom you are required to review

Think aloud
1. "Review the student as you normally would, verbalizing your thoughts along the way. I will stay quiet for the most part, though I will jot down notes to discuss after you have completed reviews of several students"
2. Aim for 3 students per site, at least one should be a student whom the member would not bring forward to the committee
3. Prompt periodically to continue "to think aloud"

Notes (observations and questions to revisit for clarification):
